# Antiphospholipid Antibodies From Women With Pregnancy Morbidity and Vascular Thrombosis Induce Endothelial Mitochondrial Dysfunction, mTOR Activation, and Autophagy

**DOI:** 10.3389/fphys.2021.706743

**Published:** 2021-11-29

**Authors:** Carlos M. Rodríguez, Manuela Velásquez-Berrío, Carolina Rúa, Marta Viana, Vikki M. Abrahams, Angela P. Cadavid, Angela M. Alvarez

**Affiliations:** ^1^Grupo Reproducción, Facultad de Medicina, Departamento de Microbiología y Parasitología, Universidad de Antioquia (UdeA), Medellín, Colombia; ^2^Grupo de Investigación en Trombosis, Facultad de Medicina, Universidad de Antioquia (UdeA), Medellín, Colombia; ^3^Grupo de Metabolismo y Función Vascular, Departamento de Química y Bioquímica, Facultad de Farmacia, Universidad San Pablo-CEU, CEU Universities, Madrid, Spain; ^4^Red Iberoamericana de Alteraciones Vasculares Asociadas a Transtornos del Embarazo (RIVATREM), Chillán, Chile; ^5^Department of Obstetrics, Gynecology and Reproductive Sciences, Yale School of Medicine, New Haven, CT, United States

**Keywords:** antiphospholipid antibodies, antiphospholipid syndrome, endothelial cell, mitochondria, mTOR, autophagy

## Abstract

Antiphospholipid syndrome (APS) is an autoimmune disease characterized by thrombosis and pregnancy morbidity (PM) obstetric events together with persistent high titers of circulating antiphospholipid antibodies (aPL). Several mechanisms that explain the development of thrombosis and PM in APS include the association of aPL with alterations in the coagulation cascade and inflammatory events. Other mechanisms disturbing cellular homeostases, such as mitochondrial dysfunction, autophagy, and cell proliferation, have been described in other autoimmune diseases. Therefore, the objective of this study was to investigate the impact of aPL from different patient populations on endothelial cell mitochondrial function, activation of the mammalian target of rapamycin (mTOR) and autophagy pathways, and cellular growth. Using an *in vitro* model, human umbilical vein endothelial cells (HUVECs) were treated with polyclonal immunoglobulin G (IgG) purified from the serum of women with both PM and vascular thrombosis (PM/VT), with VT only (VT), or with PM and non-criteria aPL (seronegative-obstetric APS, SN-OAPS). We included IgG from women with PM without aPL (PM/aPL-) and healthy women with previous uncomplicated pregnancies (normal human serum, NHS) as control groups. Mitochondrial function, mTOR activation, autophagy, and cell proliferation were evaluated by Western blotting, flow cytometry, and functional assays. IgG from women with PM/VT increased HUVEC mitochondrial hyperpolarization and activation of the mTOR and autophagic pathways, while IgG from patients with VT induced endothelial autophagy and cell proliferation in the absence of elevated mTOR activity or mitochondrial dysfunction. IgG from the SN-OAPS patient group had no effect on any of these HUVEC responses. In conclusion, aPL from women with PM and vascular events induce cellular stress evidenced by mitochondrial hyperpolarization and increased activation of the mTOR and autophagic pathways which may play a role in the pathogenesis of obstetric APS.

## Introduction

Antiphospholipid syndrome (APS) is an autoimmune disease characterized by thrombosis and/or obstetric events together with persistent high titers of circulating antiphospholipid antibodies (aPL) (Miyakis et al., 2006). Thrombosis in APS can involve different components of the vascular bed in any tissue or organ, such as arteries (coronary artery disease, ischemic stroke, and transient ischemic attack), veins (deep venous thrombosis of lower limbs or pulmonary embolism), or small vessels (catastrophic APS with episodes of thrombosis in small vessels of multiple organs causing a systemic dysfunction). Pregnancy-related morbidity in APS may include early or late gestational losses, intrauterine growth restriction, fetal demise, preterm labor, or preeclampsia. In addition to the aforementioned clinical diagnostics defined by the Sapporo criteria (Miyakis et al., 2006), there are other clinical presentations not included. These manifestations can be hematologic (thrombocytopenia and hemolytic anemia), cardiac (heart valve disease), cutaneous (livedo reticularis), renal (nephropathy), or neurologic (cognitive dysfunction not associated with stroke) (Ziporen et al., 1996; Asherson et al., 2003; Garcia and Erkan, 2018; Turrent-Carriles et al., 2018; Kolitz et al., 2019). While APS is still considered a relatively rare disorder, our understanding of its diagnosis and management is continuously advancing (Tektonidou et al., 2019). Some recent studies have estimated that the prevalence of APS is 50 per 100,000 people, and the incidence is 2.1 per 100,000 person-years without differences between men and women (Duarte-Garcia et al., 2019). The estimated frequency of aPL in thrombotic complications was reported to be 9.5% for deep vein thrombosis, 11% for myocardial infarction, and 13.5% for stroke (Andreoli et al., 2013), the latter being more associated in patients under 50 years of age (Petri, 2000). The prevalence of obstetric complications was reported to be between 6 and 50% (Andreoli et al., 2013; Alijotas-Reig et al., 2015; Cervera et al., 2015; Esteve-Valverde et al., 2016).

The prevalence of aPL in the general population ranges between 1 and 5%. However, only a minority of these individuals will develop APS (Gomez-Puerta and Cervera, 2014). Pathological aPL are a heterogeneous population of autoantibodies mainly directed against phospholipid-binding proteins such as cardiolipin (CL) and/or β2-glycoprotein I (β2GPI) (Di Simone et al., 2007). Anti-CL and anti-β2GPI aPL, in combination with lupus anticoagulant (LA), constitute the current laboratory criteria for diagnosis. However, there is a group of aPL classified as non-criteria including antithrombin, anti-phosphatidylserine, and anti-phosphatidylethanolamine antibodies which are associated with APS (Bertolaccini et al., 2011). Several studies have described patients who lack the classical clinical manifestations of APS but who present consistently with high aPL positivity, and these cases are known as “non-criteria APS” (Tektonidou et al., 2019). In contrast, patients with clinical manifestations fulfilling APS classification criteria but who are consistently negative for aPL tests are classified as “Seronegative APS” patients (Hughes and Kamashta, 2003; Jara et al., 2017; Conti et al., 2019; Hughes and Khamashta, 2019).

Several studies have described the mechanisms by which aPL lead to prothrombotic and proinflammatory states. In endothelial cells, these mechanisms include alterations in the coagulation cascade and platelet activation; increased production of reactive oxygen species (ROS) and pro-inflammatory cytokines; and decreased nitric oxide production (Hidalgo, 2014; Mulla et al., 2018; Schreiber et al., 2018). Anti-β2GPI antibodies are recognized as the most pathogenic subset of aPL. Among them, the anti-domain I β2GPI antibodies have a strong correlation with thrombosis and with pregnancy morbidity (PM) (Iwaniec et al., 2017; Liu et al., 2020), which has been used as a predictor tool for patients with late PM (Chighizola et al., 2018). Studies have demonstrated that anti-β2GPI antibodies can activate receptors such as toll-like receptor (TLR) 4, TLR2, and Apolipoprotein E receptor E2 (APOER2) expressed on the surface of endothelial cells (Ramesh et al., 2011; Benhamou et al., 2014; Raschi et al., 2014), and this can lead to the activation of the nuclear factor kappa B (NFκB), p38 mitogen-activated protein kinase (p38 MAPK), and the phosphatidylinositol 3-kinase (PI3K) signaling pathways (Meroni et al., 2014; Chighizola et al., 2015). Another mechanism involved in APS pathophysiology is oxidative stress (Alves and Grima, 2003). It was recently demonstrated that monocytes and neutrophils, from patients with APS, display increased ROS production, increased expression of pro-inflammatory and prothrombotic molecules, and a loss of mitochondria function (Perez-Sanchez et al., 2012; Lopez-Pedrera et al., 2016). This mitochondrial dysfunction was also described in a mouse model of systemic lupus erythematosus (SLE) and was associated with activation of the PI3K pathway and mammalian target of rapamycin (mTOR) (Oaks et al., 2016), a kinase that modulates cellular growth, proliferation, and apoptosis (Magnuson et al., 2012). Activation of mTOR was also increased in renal endothelial cells from patients with APS samples (Canaud et al., 2014; Chighizola et al., 2015). In addition to cell growth and survival, mTOR activation is associated with anabolic mechanisms at the intracellular level (Magnuson et al., 2012), which leads to inhibition of catabolic processes like autophagy. However, the mTOR and autophagic pathways may both be activated under conditions associated with oxidative stress and inflammation (Chen et al., 2011, 2016). Despite evidence of alterations in these pathways in other autoimmune diseases such as SLE (Lui et al., 2008; Oaks et al., 2016), less is known about the relationship between cellular metabolism and homeostasis in the context APS, and in particular, how aPL may disrupt the balance in endothelial cells. Therefore, the objective of this study was to investigate the impact of aPL on endothelial cell mitochondrial function, activation of the mTOR and autophagy pathways, and cellular growth.

## Materials and Methods

### Cell Culture

Human umbilical vein endothelial cells (HUVEC) were isolated from umbilical cords obtained from uncomplicated pregnancies based on a modified protocol by Jaffe et al. (1973) and as previously described (Velásquez et al., 2019; Gil-Villa et al., 2020). In brief, umbilical veins were perfused with 100 μg/ml type I collagenase (Invitrogen, Waltham, MA, United States) and incubated for 20 min at 37°C. Cells were recovered, and after centrifugation (50 *g* for 5 min), they were seeded in the endothelial cell growth medium (Promocell, Heidelberg, Germany) supplemented with 2% fetal bovine serum (FBS, Gibco, Waltham, MA, United States), 100 U/ml penicillin (Sigma Aldrich, Missouri, United States), 50 μg/ml gentamicin (Genfar, Bogotá, Colombia), and 0.25 μg/ml amphotericin B (Vitalis, Bogotá, Colombia). Isolated HUVECs were cultured in T75 cell culture flasks (Thermo Fisher Scientific, Waltham, MA, United States) at 37°C and 5% CO_2_ until 100% confluent. The endothelial cell phenotype (CD31+) was confirmed by flow cytometry. All experiments were performed with different HUVEC clones from passages 1–3. All treatments were performed in Opti-MEM (Gibco) to keep the cells in FBS-free conditions.

### Study Subjects

Patients were recruited from the Recurrent Pregnancy Loss Program of the Reproduction Group (University of Antioquia) and the Anticoagulation Clinic (San Vicente Fundación Hospital). Our Ethics Review Committee (Medical Investigations Institute from the School of Medicine, University of Antioquia) approved the collection of patient sera, and written consent was obtained from all participants. Women with clinical manifestations of APS were divided into the following three groups of study: women with clinical manifestations of PM and vascular thrombosis (PM/VT) or VT only (VT), positive for aPL as defined by the Sapporo criteria, and women with PM and positive for non-criteria aPL: seronegative-obstetric APS (SN-OAPS). Additionally, women with PM without aPL (PM/aPL-) and healthy women with previous uncomplicated pregnancies (normal human serum, NHS) were also included as control groups. Polyclonal immunoglobulin G (IgG) was purified from the serums of a total of 50 women included in this study for future cell treatments, and each group consisted of 10 patients. None of the patients were pregnant at the time the serum samples were obtained.

### Antiphospholipid Antibodies

Anticardiolipin antibodies (aCL) were detected using a Commercial aCL ELISA Kit (BioSystems, Barcelona, Spain). Anti-β2GPI antibodies were detected using the AESKULISA β2-Glyco-GM Kit (Aesku Diagnostics, Wendelsheim, Germany) and Imtec β2GPI Kit (Human Biochemica und Diagnostica GmbH, Magdeburg, Germany). LA was detected in plasma samples following the recommendations of the Clinical and Laboratory Standards Institute (Ratzinger et al., 2017). APTT-SP (Instrumentation Laboratory, Bedford, MA, United States) was used to demonstrate the dependence of antibodies for phospholipids. Dilute Russell’s viper venom time (dRVVT) screen and dRVVT confirmation (Instrumentation Laboratory) were used to detect LA. In addition, other non-criteria aPL were detected using an in-house ELISA standardized by the reproduction group based on the technique published by Kwak et al. (1992) and as previously described (Velásquez et al., 2019). In brief, U-bottom 96-well polystyrene microplates (Maxisorp Nunc^TM^, Thermo Fisher Scientific) were covered with 30 μl of 50 μg/ml of the following phospholipids suspended in methanol: phosphatidylglycerol, phosphatidic acid, phosphatidylserine, phosphatidylethanolamine, and phosphatidylinositol (Sigma-Aldrich, Saint Louis, MO, United States). The microplates were allowed to dry at 4°C overnight, then washed with 1 × phosphate buffered saline (PBS), and blocked with a buffer solution of PBS and 20% adult bovine serum (ABS, Gibco, United States) for 90 min at room temperature in the dark. After another wash with PBS, 50 μl of the sera or IgG of the patient were added in duplicate at a dilution of 1:50 or 250 μg/ml, respectively, in 20% ABS and incubated for 2 h in the dark. Then, the microplates were washed three times with PBS and incubated for 90 min with 50 μl of a 1:1,000 dilution of the antihuman IgG antibody conjugated to alkaline phosphatase (Thermo Fisher Scientific) and washed as above. Notably, 50 μl of the developer solution p-nitrophenyl phosphate (Sigma-Aldrich) was added at 1 mg/ml in a substrate solution (10% diethanolamine, 0.005% MgCl_2_, and 0.02% sodium azide, pH = 9.8). The reaction was stopped with 50 μl of a 3 M NaOH solution. The optical density of each well was determined using an ELISA microplate reader (Multiskan FC^TM^, Thermo Scientific) at a wavelength of 405–410 nm. In all assays, a blank with a developer solution and stop solution was included, as was a positive control and a negative control for each of the antigens. In addition, a non-specific binding control was included by placing each of the sera or IgG in a well without antigen, the value of which is subtracted from the average of the optical densities of the samples. Optical density values of the samples equal to or greater than 25% of the optical density of the positive control were considered positive. All patients were tested twice, at least 12 weeks apart.

To purify the total polyclonal IgG from the patient sera for the subsequent treatment of HUVECs, affinity chromatography was performed as previously described (Alvarez et al., 2017) using a MAb Trap^TM^ Kit (GE Healthcare, Chicago, IL, United States). In brief, serum samples from each group were pooled, and the total protein was quantified. Pooled samples were centrifuged, filtered, and diluted 1:1 with a binding buffer to load samples of up to 25 mg. Samples were passed through a protein G Sepharose^®^ prepacked column and eluted with the buffer supplied. The purified IgG was tested for endotoxins using the Limulus Amebocyte Lysate QCL-1000^TM^ Kit (Lonza, Basilea, Swiss), and all preparations tested negative (data not shown). IgG integrity was also checked by performing sodium dodecyl sulfate-polyacrylamide gel electrophoresis (SDS-PAGE) under reduced and non-reduced conditions (data not shown).

### Mitochondrial Membrane Potential and Lysosomal Acidification

Human umbilical vein endothelial cells were cultured in 24 well plates (5 × 10^4^ cells/well) at 37°C and 5% CO_2_. After 24 h, cells were incubated with 250 μg/ml IgG from all groups for a further 24 h. Then, cells were trypsinized, centrifuged, and stained either with 0.003 ng/ml 3,3′-dihexyloxacarbocyanine iodide (DiOC-6) (Thermo Fisher Scientific) and 0.06 ng/ml propidium iodide (PI) (Sigma-Aldrich) for the mitochondrial membrane potential (MMP) test or with LysoTracker green DND-26 TM (Thermo Fisher Scientific) for the lysosomal acidification test. Then, flow cytometry was performed using an LSR Fortessa (Becton Dickinson, Franklin Lakes, NJ, United States), and at least 10,000 events per sample were acquired. The median fluorescence intensity (MFI) was recorded for DiOC-6 and LysoTracker green.

### Western Blot

Whole-cell lysates were prepared from HUVECs grown in six well plates (1 × 10^5^ cells/well) and stimulated with 250 μg/ml IgG for 1–24 h using 120 μl Laemmli sample buffer (Tris 1 M pH = 6.8, 20% SDS, 20% glycerol, 3.8% β-mercaptoethanol, and 8% bromophenol blue). Notably, 40 μl of protein extracts were resolved on 8–15% SDS-PAGE gels. To detect LC3-II/LC3-I, 30% glycerol was added to the gels. Separated proteins were transferred to the polyvinylidene difluoride (PVDF) membrane (Amresco, Solon, OH, United States). After blocking with 5% non-fat dry milk, membranes were incubated overnight at 4°C with 1:1,000 dilution of the following primary antibodies in 10% bovine serum albumin (Thermo Fisher Scientific): rabbit antihuman phospho-RPS6 (Ser235/236), rabbit antihuman total RPS6, rabbit antihuman phospho-ULK1 (Ser757), rabbit antihuman total ULK1, rabbit antihuman LC3-I and LC3-II, and mouse antihuman α-tubulin (Cell Signaling Technology, Beverly, MA, United States). Membranes were washed and incubated with 1:2,000 or 1:5,000 dilution of goat anti-rabbit or anti-mouse IgG secondary antibodies conjugated to horseradish peroxidase (Cell Signaling Technology). Peroxidase conjugated antibodies were detected by chemiluminescence using SuperSignal West Pico (Thermo Fisher Scientific). Images were captured using a G-Box photodocumentator (Syngene, Cambridge, England), and densitometry analysis was performed using Image J 1.51 (NIH, Bethesda, MA, United States).

### Proliferation Assay

Human umbilical vein endothelial cells at 1 × 10^3^ were seeded into 96 well microplates and cultured for 24 h. Then, cells were treated with 250 μg/ml IgG for a further 24 h. Cell proliferation was measured using the BrdU Cell Proliferation Assay Kit (Cell Signaling Technology) following the instructions of the manufacturer. Optical densities were read at 450 nm on a Multiskan FC plate reader (Thermo Fisher Scientific). Optical density was directly proportional to the number of proliferating cells.

### Statistical Analysis

All experiments were performed at least three times. Data are expressed as mean ± SE of the mean (SEM). Statistical significance was determined using one-way ANOVA with Holm-Šídák or Dunns post-test according to the data distribution, using GraphPad Prism 6^TM^ (GraphPad Software Inc., La Joya, CA, United States).

## Results

### Characteristics of Women Included in This Study

Women from the PM/VT and VT groups presented with clinical and laboratory features in keeping with the Sapporo criteria. The group of SN-OAPS women had a history of pregnancy-related morbidity, but they were only positive for the non-criteria aPL such as anti-phosphatidylglycerol and anti-phosphatidylethanolamine. The control groups, namely, PM/aPL- and healthy NHS women, were negative for all aPL laboratory tests. As expected, women from the PM/VT and VT presented with significantly higher levels of anti-β2GPI and anti-CL antibody titers when compared with the NHS and PM/aPL- groups. Data from the clinical and laboratory analyses are presented in Table 1.

**TABLE 1:** Clinical and laboratory features of the women included.

**Parameter**		**NHS (*n* = 10)**	**PM/aPL- (*n* = 10)**	**PM/VT (*n* = 10)**	**SN-OAPS (*n* = 10)**	**VT (*n* = 10)**
Age (mean of years ± SD)		37.6 ± 7.6	30.9 ± 5.5	36.2 ± 5.8	32.5 ± 4.6	32.8 ± 9.9
Previous fetal losses (mean and range) ≤ 10 weeks of pregnancy > 10 weeks of pregnancy		0 0	1.9 (1–3) 0.7 (1–3)	1.3 (1–5) 1.7 (1–5)	1.2 (1–2) 0.9 (1–4)	0 0
Preeclampsia < 34 weeks, n		0	0	6	0	0
Intrauterine growth restriction, n		0	0	2	0	0
Venous/arterial thrombosis, n		0	0	10	0	6
Associated systemic rheumatic autoimmune disease, n		0	0	1	0	4
Lupus anticoagulant (mean ± SD) ^£^		1.06 ± 0.12	1.02 ± 0.08	2.43 ± 0.8 (+)^a,b^	1.06 ± 0.07	2.62 ± 0.55 (+)^a,b^
Positive patients for lupus anticoagulant, *n*		0	0	9	0	10
IgG aβ2GPI in serum (U/mL)^†^		2.29 ± 0.07	2.82 ± 0,43	73.4 ± 82 (+)^c,d^	3.16 ± 0.26	21.27 ± 30.5 (+)
Positive patients for IgG aβ2GPI, n		0	0	9	0	7
IgG anti-cardiolipin in serum (GPL/mL) ^‡^		0	2.31 ± 0.45	113 ± 92.8 (+)^c,d^	2.05 ± 0.61	44.3 ± 43.9 (+)
Positive patients for IgG anti-cardiolipin		0	0	9	0	8
aβ2GPI in 250 μg/mL of IgG purified from sera (U/mL)^§^		0	0	62.75 (+)	0	27.21 (+)
Anti-cardiolipin in 250 μg/mL of IgG purified from sera (GPL/mL)^‡^		4.95	3.85	82.9 (+)	4.97	21.8 (+)
Serum other no-criteria IgG antiphospholipid antibodies (percentage of positivity of mean OD of patients/mean OD of positive controls)^*^ [number of positive patients]	aPG	2.87 ± 5.07 [0]	3.62 ± 6.31 [0]	76.62 ± 57.89 (+) [8]	65.36 ± 57.0 (+) [7]	24.5 ± 32.91 (+) [3]
	aPA	4.38 ± 5.68 [0]	1.65 ± 1.79 [0]	62.91 ± 53.16 (+) [7]	26.36 ± 21.08 (+) [5]	46.79 ± 60.83 (+) [4]
	aPS	2.23 ± 2.56 [0]	6.53 ± 7.81 [0]	87.76 ± 112.23 (+) [6]	55.84 ± 52.07 (+) [7]	45.82 ± 41.24 (+) [4]
	aPE	1.28 ± 3.34 [0]	5.42 ± 6.22 [0]	28.10 ± 33.73 [4]	44.80 ± 36.39 (+) [6]	37.29 ± 52.97 (+) [5]
	aPI	5.51 ± 4.08 [0]	4.23 ± 5.23 [0]	56.19 ± 46.1 (+) [7]	48.39 ± 28.11 (+) [9]	42.88 ± 42.85 (+) [4]

n, number of patients; (+) positive result; ^*a*^*p* < 0.001 vs. NHS; ^*b*^*p* < 0.001 vs. PM/aPL-; ^*c*^*p* < 0.05 vs. NHS; ^*d*^*p* < 0.05 vs. PM/aPL-; £ Positive > 1.2; ^†^Positive at > 15 U/ml; ^‡^Positive at > 10 GPL; ^§^ Positive at > 7 U/ml; * Positive at > 25%; aPG, anti-phosphatidylglycerol antibodies; aPA, anti-phosphatidic acid antibodies; aPS, anti-phosphatidyl serine antibodies, aPE, anti-phosphatidyl ethanolamine antibodies; aPI, anti-phosphatidyl inositol antibodies; aPL, antiphospholipid antibodies; NHS, normal human serum; PM, pregnancy morbidity; VT, vascular thrombosis; SN-OAPS, seronegative obstetric antiphospholipid syndrome; SD, standard deviation, IgG, Immunoglobulin G; aβ2GPI, anti-β2glicoprotein I; AL, lupus anticoagulant; GPL, standard units of IgG anticardiolipin.

### Immunoglobulin G From Women With Pregnancy Morbidity/Vascular Thrombosis Increase Endothelial Cell Mitochondrial Membrane Potential

**FIGURE 1:**
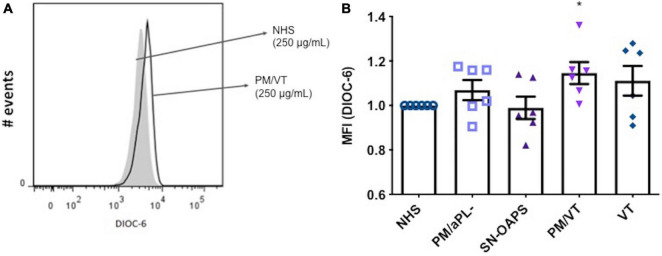
Mitochondrial membrane potential (MMP) of endothelial cells is increased by antiphospholipid antibodies (aPL). Human umbilical vein endothelial cells (HUVECs) were stimulated with polyclonal immunoglobulin G (IgG) (250 μg/ml) from women with clinical features of antiphospholipid syndrome (APS) [seronegative-obstetric APS (SN-OAPS); pregnancy morbidity and vascular thrombosis (PM/VT); and VT] and control groups [normal human serum (NHS) and PM/aPL-]. MMP was measured by 3,3′-dihexyloxacarbocyanine iodide(DiOC-6) incorporation and flow cytometric analysis. **(A)** Representative histogram showing HUVECs treated with NHS IgG and PM/VT IgG. **(B)** The chart shows that treatment of HUVECs with PM/VT IgG induced a significant increase in MMP in comparison with NHS IgG. *n* = 6; *p < 0.05 vs. NHS as determined by one-way ANOVA and Dunn’s post-test.

The balance between proton pumping by the electron transport chain and proton flow by complex V determines the MMP of a cell (Zorova et al., 2018; Morganti et al., 2019). High MMP levels result in the activation of the mitochondrial respiratory chain, which is an important source of ROS. Since high levels of ROS can cause cellular injury, maintaining elevated MMP levels is potentially harmful (Zorova et al., 2018). As shown in Figure 1, IgG from women in the PM/VT group significantly increased HUVEC MMP levels when compared with the NHS control. Levels of HUVEC MMP in response to IgG from patients with PM/aPL- were similar to that after exposure to the NHS control (Figure 1). No significant differences in HUVEC MMP levels were found after treatment with IgG from the PM/VT or SN-OAPS groups when compared with the NHS control group (Figure 1).

### Immunoglobulin G From Women With Pregnancy Morbidity/Vascular Thrombosis Activate the Mammalian Target of Rapamycin Pathway in Endothelial Cells

**FIGURE 2:**
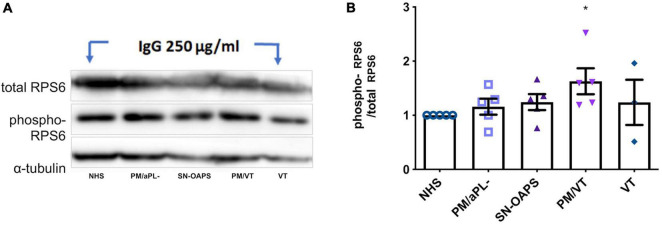
Activation of mammalian target of rapamycin (mTOR) pathway is induced by aPL in endothelial cells. HUVECs were stimulated with 250 μg/ml polyclonal IgG from women with clinical features of APS and control groups. Protein expression of the total and phosphorylated forms of the effector ribosomal protein S6 (RPS6) was assessed by Western blot. **(A)** Representative blot. **(B)** Scatter plot shows the phospho/total RPS6 ratio. PM/VT IgG increased the RPS6 phosphorylation in comparison with the NHS IgG, which means that activates the mTOR pathway. Data are shown as mean ± SEM (*n* = 5; *p < 0.05 vs. NHS from one-way ANOVA and Dunn’s post-test).

Perturbation of mitochondrial function and subsequent ROS production is known to influence the activation of the mTOR pathway, which in turn can impact the mitochondria through a retrograde signaling pathway (Kim et al., 2002; Sarbassov et al., 2005; Hopper et al., 2006; Schieke et al., 2006; Wullschleger et al., 2006). Therefore, we then evaluated the activity of the mTOR pathway by measuring phosphorylation of its effector protein RPS6. As a control, the mTOR inhibitor, rapamycin, reduced HUVEC phospho-RPS6 expression (Supplementary Figure 1). HUVEC expression levels of phospho-RPS6 after normalization to total RPS6 were similar in response to IgG from the NHS and PM/aPL- control groups (Figure 2). IgG from the PM/VT group significantly increased HUVEC phospho-RPS6 expression in comparison with IgG from the NHS control group (Figure 2). IgG from the VT and SN-OAPS groups had no significant effect on HUVEC phospho-RPS6 expression in comparison with either the NHS or the PM/aPL- controls (Figure 2).

### Immunoglobulin G From Women With Pregnancy Morbidity/Vascular Thrombosis and Vascular Thrombosis Induces Autophagy in Endothelial Cells

**FIGURE 3:**
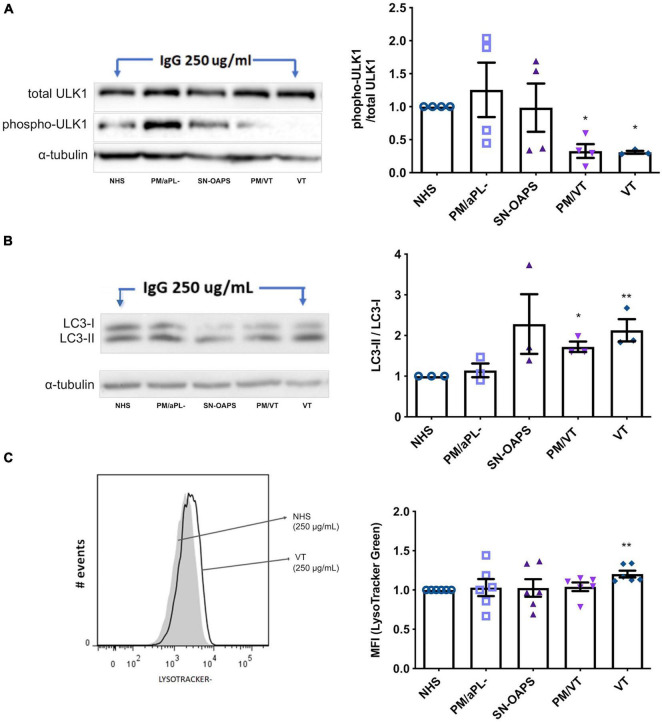
Autophagy is activated by aPL in endothelial cells. HUVECs were stimulated with polyclonal IgG (250 μg/ml) from women with clinical features of APS (SN-OAPS; PM/VT; and VT) and control groups (NHS and PM/aPL-). **(A,B)** The early autophagy marker ULK1 and the late autophagy marker LC3-I/LC3-II were evaluated by Western blot. **(A)** Representative blot for phosphorylated and total ULK1. The chart shows the phospho/total ULK1 ratio. PM/VT and VT IgG significantly reduced ULK1 phosphorylation in comparison with NHS IgG (*n* = 4; *p < 0.05 vs. NHS as determined by one-way ANOVA and Dunn’s post-test). **(B)** Representative blot for LC3-I and LC3-II. The chart shows the LC3-II-LC3-I ratio. PM/VT and VT IgG significantly increased the LC3-II-LC3-I ratio in comparison with NHS IgG (*n* = 3; *p < 0.05 and **p < 0.01 vs. NHS as determined by one-way ANOVA and Holm-Sidak’s post-test). **(C)** To evaluate autophagy at a functional level, lysosomal acidification was assessed using LisoTracker Green and flow cytometric analysis. Representative histogram showing HUVECs treated with NHS IgG and VT IgG. The chart shows the levels of LisoTracker Green as median fluorescence intensity (MFI) and that treatment of HUVECs with VT IgG significantly increased lysosomal acidification. *n* = 6; **p < 0.01 vs. NHS as determined by one-way ANOVA and Dunn’s post-test.

To examine whether aPL have an effect on endothelial cell autophagy, we performed Western blots for the early autophagy marker ULK1 and the late autophagy marker LC3-II/LC3-I (Yu et al., 2018). As a control, the autophagy inducer, rapamycin, reduced HUVEC phospho-ULK1 and increased LC3-II/LC3-I levels (Supplementary Figures 2, 3). HUVEC expression levels of phospho-ULK1 after normalization to total ULK-1 were similar in response to IgG from the NHS and PM/aPL- control groups (Figure 3A). IgG from groups PM/VT and VT significantly reduced HUVEC ULK1 phosphorylation in comparison with the NHS control, while there was no significant difference with IgG from the SN-OASP group (Figure 3A). Similarly, the LC3-II-LC3-I ratio was significantly increased in HUVECs exposed to IgG from the PM/VT and VT groups when compared with the NHS control, while IgG from the SN-OASP group or the PM/aPL- control had no effect (Figure 3B). To further investigate autophagy at a functional level, HUVEC lysosomal acidification was examined (Yim and Mizushima, 2020) using LysoTracker^®^. As a positive control, rapamycin induced an increase in HUVEC lysosomal acidification, when compared to untreated cells (Supplementary Figure 4). As shown in Figure 3C, IgG from the VT group significantly increased HUVEC lysosomal acidification when compared with the NHS control. However, there was no evidence of increased HUVEC lysosomal acidification with IgG from the other groups (Figure 3C).

### Immunoglobulin G From Women With Vascular Thrombosis Increases Endothelial Cell Proliferation

**FIGURE 4:**
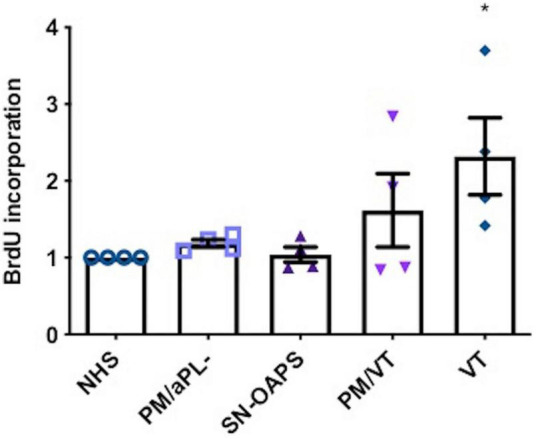
Endothelial cell proliferation is increased by aPL. HUVECs were stimulated with polyclonal IgG (250 μg/ml) from women with clinical features of APS (SN-OAPS; PM/VT; and VT) and control groups (NHS and PM/aPL-). Cell proliferation was measured by BrdU incorporation. The chart shows that VT IgG significantly increased cell proliferation in comparison with NHS IgG. *n* = 4; *p < 0.05 vs. NHS as determined by one-way ANOVA and Holm-Sidak’s post-test.

Since the main cellular processes controlled by the mTOR pathway are cell growth, proliferation, and survival, we evaluated the effect of IgG from the patient groups on endothelial cell proliferation using a BrdU incorporation assay. Levels of HUVEC cell proliferation were similar in the presence of IgG from the control groups NHS and PM/aPL-. IgG from individuals with VT significantly increased HUVEC proliferation in comparison with NHS only, while there was no effect by IgGs from the PM/VT or the SN-OAPS groups (Figure 4).

## Discussion

Endothelial cells are responsible for maintaining vascular homeostasis and play an important role in the development of thrombosis in patients with APS (Poredos and Jezovnik, 2018). These cells are the target of several bioactive circulating factors that can cause a generalized dysfunction through deregulations in metabolism (Possomato-Vieira and Khalil, 2016; Bierhansl et al., 2017). MMP plays a key role in the maintenance of mitochondrial homeostasis (Zorova et al., 2018). MMP of a cell can change depending on the microenvironment, access to nutrients, cellular stress, and metabolic activity (Hirata and Sahai, 2017). aPL can induce a perturbation of the MMP in trophoblast cells, hepatocytes, and lymphocytes, leading to depolarization or hyperpolarization (Lai et al., 2015; Oaks et al., 2016; Alvarez et al., 2017). Since both depolarization and hyperpolarization involve dissociation of the electron transport chain and an increase of oxygen consumption, these conditions can be harmful to the cell through the induction of oxidative or reductive stress (Guo et al., 2011; Oaks et al., 2016; Zorova et al., 2018). We previously reported that serum from women with PM/VT increased the intracellular and mitochondrial production of ROS in HUVECs (Velásquez et al., 2019), the same group of patients whose IgGs induced the high MMP (hyperpolarization) in this study. Taken together, these findings suggest a mechanism by which aPL from women with PM/VT induced HUVEC oxidative stress. In fact, oxidative stress has been associated with endothelial cell dysfunction in preeclampsia, a clinical manifestation of obstetric APS (Rodríguez-Almaraz et al., 2018). Moreover, in the trophoblast, aPL recognizing CL and β2GPI bind to the mitochondria and induce ROS production (Zussman et al., 2020).

Oxidative cellular stress resulting from excessive metabolic ROS production can lead to the activation of rescue mechanisms such as autophagy. Autophagy is an intracellular degradation pathway that traffics substrates that could otherwise cause cytotoxicity (Chen et al., 2009; Ryter et al., 2019) through a catabolic system of double-membrane vesicles called autophagosomes, which are then fused with lysosomes (Bento et al., 2016; Qian et al., 2017). While some studies have reported altered autophagy in experimental models of APS (Mulla et al., 2018; Mu et al., 2020), less is known about how aPL impact endothelial cell autophagy. Endothelial cells control vascular homeostasis (Badimón and Martínez-González, 2002), and the mTOR pathway plays a major role in regulating cell metabolism, growth, and survival. There is also cross talk between the mTOR and autophagic pathways (Ryter et al., 2019). Classically, activation of the mTOR pathway has been associated with inhibition of autophagy through phosphorylation of ULK1 protein at Ser757 (Kim et al., 2011). However, in other contexts, such as tumor growth, there is coexistence between mTOR and autophagy activation. A similar behavior was observed in this study since the mTOR activation (determined by RPS6 phosphorylation) and concomitant autophagy activity (determined by a loss of ULK1 phosphorylation and increased LC3-II/LC3-I expression) were induced by IgG from patients with PM/VT and also IgG from patients with VT. In addition, IgG from women of the VT group induced lysosomal acidification, the ability of these aPL to trigger the final step of this degradative/recycling pathway.

Augmented endothelium cell survival has been associated with pathological conditions since it can lead to hyperplasia resulting in the occlusion of vessels and thus, generate a prothrombotic environment (Rajendran et al., 2013; Widlansky and Malik, 2015). aPL-induced proliferation was previously demonstrated in trophoblast cells (Alvarez et al., 2017). Another study showed increased cell proliferation in vascular endothelial cells exposed to aPL, which was associated with mTOR activation; and this activation was also observed in renal microvasculature of patients with APS nephropathy. In addition, patients who received rapamycin showed decreased vascular proliferation and no recurrence of vascular lesions (Canaud et al., 2014). This study highlights the relationship between cell survival pathways and cell proliferation under aPL conditions. Our findings of elevated cell proliferation in response to IgG from patients with VT only are, in part, in agreement with this report and indicate a link between the thrombotic features of that group of patients and the aPL-induced vascular effects.

## Conclusion

This study aimed to establish a link between endothelial cell mitochondrial dysfunction, mTOR activation, and autophagy in the context of aPL, although it was performed in a descriptive way. Our findings have shown endothelial cell mitochondrial dysfunction in association with activation of the mTOR pathway and concurrent autophagic activity in response IgG from patients with PM/VT, while IgG from patients with VT only induced endothelial autophagy and cell proliferation in the absence of elevated mTOR activity or mitochondrial dysfunction. This underscores the heterogeneity of aPL. As expected, IgG from the control group PM/aPL- did not induce any responses in the endothelial cells. IgG from the patient group with clinical features of PM but non-criteria aPL (SN-OAPS) also did not induce any responses in the endothelial cells, thus revealing specific mechanisms triggered by the classical pathological aPL present in patients with PM/VT and VT. We previously showed several *in vitro* effects induced by aPL from women with PM/VT when compared with aPL from women with PM alone, suggesting that these aPL are distinct and could be triggering other pathways, which leads to different and/or more complex clinical manifestations (Alvarez et al., 2015, 2017). These subtle differences among the mechanisms triggered by aPL subpopulations have been suggested from prior studies (Ripoll et al., 2018; Alvarez et al., 2021). We also highlight that our cellular model was FBS-free, and since we did not performed any recombinant β2GPI addition, it is possible that cellular responses observed here were induced by aCL rather than aB2GPI antibodies. In conclusion, aPL from women with PM and vascular events induce cellular stress evidenced by mitochondrial hyperpolarization and increased activation of the mTOR and autophagic pathways, which may play a role in the pathogenesis of obstetric APS. These pathways may provide us with pharmacological targets to study further since compounds such as rapamycin and chloroquine that inhibit mTOR and autophagy have been used in experimental models of APS (Xia et al., 2017; Liu et al., 2019; Miranda et al., 2019).

## Data Availability Statement

The raw data supporting the conclusion of this article will be made available by the authors, without undue reservation.

## Ethics Statement

The studies involving human participants were reviewed and approved by Ethics Committee from the Medical Investigations Institute from the School of Medicine (University of Antioquia). The patients/participants provided their written informed consent to participate in this study.

## Author Contributions

AA and AC responsible for obtaining the funds and designed this study. CRú and AC were responsible for the recruitment of patients. CRo and MV-B performed the experiments and analyzed the data. CRú performed and analyzed the hematological tests. CRo, AA, AC, MV, and VA wrote and performed a critical review of the manuscript. All the authors contributed to interpreting the results and revising the manuscript.

## Conflict of Interest

The authors declare that the research was conducted in the absence of any commercial or financial relationships that could be construed as a potential conflict of interest.

## Publisher’s Note

All claims expressed in this article are solely those of the authors and do not necessarily represent those of their affiliated organizations, or those of the publisher, the editors and the reviewers. Any product that may be evaluated in this article, or claim that may be made by its manufacturer, is not guaranteed or endorsed by the publisher.

## References

[B1] Alijotas-Reig J. Ferrer-Oliveras R. Ruffatti A. Tincani A. Lefkou E. Bertero M. T. (2015). The European Registry on Obstetric Antiphospholipid Syndrome (EUROAPS): a survey of 247 consecutive cases. *Autoimmun. Rev.* 14 387–395. 10.1016/j.autrev.2014.12.010 25555817

[B2] Alvarez A. M. Balcázar N. San Martín S. Markert U. R. Cadavid A. P. (2017). Modulation of antiphospholipid antibodies-induced trophoblast damage by different drugs used to prevent pregnancy morbidity associated with antiphospholipid syndrome. *Am. J. Reprod. Immunol.* 77:e12634. 10.1111/aji.12634 28132398

[B3] Alvarez A. M. Gomez-Gutierrez A. M. Bueno-Sanchez J. C. Rua-Molina C. Cadavid A. P. (2021). Obstetric antiphospholipid syndrome: an approach from glycans of the immunoglobulin G. *J. Hum. Reprod. Sci.* 14 97–100. 10.4103/jhrs.JHRS_21_20 34084001 PMC8057144

[B4] Alvarez A. M. Mulla M. J. Chamley L. W. Cadavid A. P. Abrahams V. M. (2015). Aspirin-triggered lipoxin prevents antiphospholipid antibody effects on human trophoblast migration and endothelial cell interactions. *Arthr. Rheumatol.* 67 488–497. 10.1002/art.38934 25370166

[B5] Alves J. D. Grima B. (2003). Oxidative stress in systemic lupus erythematosus and antiphospholipid syndrome: a gateway to atherosclerosis. *Curr. Rheumatol. Rep.* 5 383–390. 10.1007/s11926-003-0029-1 12967525

[B6] Andreoli L. Chighizola C. B. Banzato A. Pons-Estel G. J. Ramire de Jesus G. Erkan D. (2013). Estimated frequency of antiphospholipid antibodies in patients with pregnancy morbidity, stroke, myocardial infarction, and deep vein thrombosis: a critical review of the literature. *Arthr. Care Res.* 65 1869–1873. 10.1002/acr.22066 23861221

[B7] Asherson R. A. Cervera R. de Groot P. G. Erkan D. Boffa M. C. Piette J. C. (2003). Catastrophic antiphospholipid syndrome: international consensus statement on classification criteria and treatment guidelines. *Lupus* 12 530–534. 10.1191/0961203303lu394oa 12892393

[B8] Badimón L. Martínez-González J. (2002). Endotelio en la protección vascular: nuevos conocimientos. *Rev. Española Cardiol.* 55 17–26. 15626352

[B9] Benhamou Y. Bellien J. Armengol G. Brakenhielm E. Adriouch S. Iacob M. (2014). Role of Toll-like receptors 2 and 4 in mediating endothelial dysfunction and arterial remodeling in primary arterial antiphospholipid syndrome. *Arthritis Rheumatol.* 66 3210–3220. 10.1002/art.38785 25047402

[B10] Bento C. F. Renna M. Ghislat G. Puri C. Ashkenazi A. Vicinanza M. (2016). Mammalian Autophagy: how Does It Work? *Annu. Rev. Biochem.* 85 685–713. 10.1146/annurev-biochem-060815-014556 26865532

[B11] Bertolaccini M. Amengual O. Atsumi T. Binder W. L. Laat B. D. Forastiero R. (2011). Non-criteria’aPL tests report of a task force and preconference workshop at the 13th International Congress on Antiphospholipid Antibodies Galveston, TX, USA, April 2010. 20 191–205. 10.1177/0961203310397082 21303836

[B12] Bierhansl L. Conradi L. C. Treps L. Dewerchin M. Carmeliet P. (2017). Central Role of Metabolism in Endothelial Cell Function and Vascular Disease. *Physiology* 32 126–140. 10.1152/physiol.00031.2016 28202623 PMC5337830

[B13] Canaud G. Bienaimé F. Tabarin F. Bataillon G. Seilhean D. Noël L.-H. (2014). Inhibition of the mTORC pathway in the antiphospholipid syndrome. *N. Engl. J. Med.* 371 303–312. 25054716 10.1056/NEJMoa1312890

[B14] Cervera R. Serrano R. Pons-Estel G. J. Ceberio-Hualde L. Shoenfeld Y. de Ramon E. (2015). Morbidity and mortality in the antiphospholipid syndrome during a 10-year period: a multicentre prospective study of 1000 patients. *Ann. Rheum. Dis.* 74 1011–1018. 10.1136/annrheumdis-2013-204838 24464962

[B15] Chen D. Liu J. Lu L. Huang Y. Wang Y. Wang M. (2016). Emodin attenuates TNF-α-induced apoptosis and autophagy in mouse C2C12 myoblasts though the phosphorylation of Akt. *Int. Immunopharmacol.* 34 107–113. 10.1016/j.intimp.2016.02.023 26943728

[B16] Chen L. Xu B. Liu L. Luo Y. Zhou H. Chen W. (2011). Cadmium induction of reactive oxygen species activates the mTOR pathway, leading to neuronal cell death. *Free Rad. Biol. Med.* 50 624–632. 10.1016/j.freeradbiomed.2010.12.032 21195169 PMC3032035

[B17] Chen Y. Azad M. Gibson S. (2009). Superoxide is the major reactive oxygen species regulating autophagy. *Cell Death Diff.* 16:1040. 10.1038/cdd.2009.49 19407826

[B18] Chighizola C. B. Pregnolato F. Andreoli L. Bodio C. Cesana L. Comerio C. (2018). Beyond thrombosis: anti-beta2GPI domain 1 antibodies identify late pregnancy morbidity in anti-phospholipid syndrome. *J. Autoimmun.* 90 76–83. 10.1016/j.jaut.2018.02.002 29454510

[B19] Chighizola C. B. Raschi E. Borghi M. O. Meroni P. L. (2015). Update on the pathogenesis and treatment of the antiphospholipid syndrome. *Curr. Opin. Rheumatol.* 27 476–482. 10.1097/BOR.0000000000000200 26125104

[B20] Conti F. Andreoli L. Crisafulli F. Mancuso S. Truglia S. Tektonidou M. G. (2019). Does seronegative obstetric APS exist? “pro” and “cons”. *Autoimmun. Rev.* 18:102407. 10.1016/j.autrev.2019.102407 31639518

[B21] Di Simone N. Luigi M. P. Marco D. Fiorella D. N. Silvia D. Clara D. M. (2007). Pregnancies complicated with antiphospholipid syndrome: the pathogenic mechanism of antiphospholipid antibodies: a review of the literature. *Ann. N. Y. Acad. Sci.* 1108 505–514. 10.1196/annals.1422.054 17894016

[B22] Duarte-Garcia A. Pham M. M. Crowson C. S. Amin S. Moder K. G. Pruthi R. K. (2019). The Epidemiology of Antiphospholipid Syndrome: a Population-Based Study. *Arthritis Rheumatol.* 71 1545–1552. 10.1002/art.40901 30957430 PMC6717037

[B23] Esteve-Valverde E. Ferrer-Oliveras R. Alijotas-Reig J. (2016). Obstetric antiphospholipid syndrome. *Rev. Clin. Esp.* 216 135–145. 10.1016/j.rce.2015.09.003 26603476

[B24] Garcia D. Erkan D. (2018). Diagnosis and Management of the Antiphospholipid Syndrome. *N. Engl. J. Med.* 378 2010–2021. 10.1056/NEJMra1705454 29791828

[B25] Gil-Villa A. M. Alvarez A. M. Velasquez-Berrio M. Rojas-Lopez M. Cadavid J. A. (2020). Role of aspirin-triggered lipoxin A4, aspirin, and salicylic acid in the modulation of the oxidative and inflammatory responses induced by plasma from women with pre-eclampsia. *Am. J. Reprod. Immunol.* 83:e13207. 10.1111/aji.13207 31696583

[B26] Gomez-Puerta J. A. Cervera R. (2014). Diagnosis and classification of the antiphospholipid syndrome. *J. Autoimmun.* 4 20–25. 10.1016/j.jaut.2014.01.006 24461539

[B27] Guo J. Y. Chen H.-Y. Mathew R. Fan J. Strohecker A. M. Karsli-Uzunbas G. (2011). Activated Ras requires autophagy to maintain oxidative metabolism and tumorigenesis. *Genes Dev.* 25 460–470. 10.1101/gad.2016311 21317241 PMC3049287

[B28] Hidalgo L. G. (2014). Inhibition of the mTORC pathway in the antiphospholipid syndrome. *N. Engl. J. Med.* 371:1554. 10.1056/NEJMc1410247 25317880

[B29] Hirata E. Sahai E. (2017). Tumor microenvironment and differential responses to therapy. *Cold Spring Harbor Perspect. Med.* 7:a026781. 10.1101/cshperspect.a026781 28213438 PMC5495051

[B30] Hopper R. K. Carroll S. Aponte A. M. Johnson D. T. French S. Shen R. F. (2006). Mitochondrial matrix phosphoproteome: effect of extra mitochondrial calcium. *Biochemistry* 45 2524–2536. 10.1021/bi052475e 16489745 PMC1415274

[B31] Hughes G. R. V. Kamashta M. A. (2003). Seronegative antiphospholipid syndrome. *Ann. Rheum. Dis.* 62:1127. 10.1136/ard.2003.006163 14644846 PMC1754381

[B32] Hughes G. R. V. Khamashta M. A. (2019). ‘Seronegative antiphospholipid syndrome’: an update. *Lupus* 28 273–274. 10.1177/0961203319826358 30691344

[B33] Iwaniec T. Kaczor M. P. Celinska-Lowenhoff M. Polanski S. Musial J. (2017). Clinical significance of anti-domain 1 beta2-glycoprotein I antibodies in antiphospholipid syndrome. *Thromb. Res.* 153 90–94. 10.1016/j.thromres.2017.02.019 28363116

[B34] Jaffe E. A. Nachman R. L. Becker C. G. Minick C. R. (1973). Culture of human endothelial cells derived from umbilical veins. Identification by morphologic and immunologic criteria. *J. Clin. Invest.* 52 2745–2756. 10.1172/JCI107470 4355998 PMC302542

[B35] Jara L. J. Medina G. Cruz-Cruz P. Olivares-Rivera J. Duarte-Salazar C. Saavedra M. A. (2017). Non-criteria or seronegative obstetric antiphospholipid syndrome. *Isr. Med. Assoc. J.* 19 382–386. 28647939

[B36] Kim D. H. Sarbassov D. D. Ali S. M. King J. E. Latek R. R. Erdjument-Bromage H. (2002). mTOR interacts with raptor to form a nutrient-sensitive complex that signals to the cell growth machinery. *Cell* 110 163–175. 10.1016/s0092-8674(02)00808-5 12150925

[B37] Kim J. Kundu M. Viollet B. Guan K.-L. (2011). AMPK and mTOR regulate autophagy through direct phosphorylation of Ulk1. *Nat. Cell Biol.* 13:132. 10.1038/ncb2152 21258367 PMC3987946

[B38] Kolitz T. Shiber S. Sharabi I. Winder A. Zandman-Goddard G. (2019). Cardiac Manifestations of Antiphospholipid Syndrome With Focus on Its Primary Form. *Front. Immunol.* 10:941. 10.3389/fimmu.2019.00941 31134062 PMC6522847

[B39] Kwak J. Y. Gilman-Sachs A. Beaman K. D. Beer A. E. (1992). Autoantibodies in women with primary recurrent spontaneous abortion of unknown etiology. *J. Reprod. Immunol.* 22 15–31. 10.1016/0165-0378(92)90003-m 1522562

[B40] Lai Z.-W. Marchena-Mendez I. Perl A. (2015). Oxidative stress and Treg depletion in lupus patients with anti-phospholipid syndrome. *Clin. Immunol.* 158 148–152. 10.1016/j.clim.2015.03.024 25862984 PMC4464983

[B41] Liu L. Q. Wang S. B. Shao Y. F. Shi J. N. Wang W. Chen W. Y. (2019). Hydroxychloroquine potentiates the anti-cancer effect of bevacizumab on glioblastoma via the inhibition of autophagy. *Biomed. Pharmacother.* 118:109339. 10.1016/j.biopha.2019.109339 31545270

[B42] Liu T. Gu J. Wan L. Hu Q. Teng J. Liu H. (2020). Anti-beta2GPI domain 1 antibodies stratify high risk of thrombosis and late pregnancy morbidity in a large cohort of Chinese patients with antiphospholipid syndrome. *Thromb. Res.* 185 142–149. 10.1016/j.thromres.2019.11.029 31816554

[B43] Lopez-Pedrera C. Barbarroja N. Jimenez-Gomez Y. Collantes-Estevez E. Aguirre M. A. Cuadrado M. J. (2016). Oxidative stress in the pathogenesis of atherothrombosis associated with anti-phospholipid syndrome and systemic lupus erythematosus: new therapeutic approaches. *Rheumatology* 55 2096–2108. 10.1093/rheumatology/kew054 27018059

[B44] Lui S. L. Tsang R. Chan K. W. Zhang F. Tam S. Yung S. (2008). Rapamycin attenuates the severity of established nephritis in lupus-prone NZB/W F1 mice. *Nephrol. Dialy. Transpl.* 23 2768–2776. 10.1093/ndt/gfn216 18445640

[B45] Magnuson B. Ekim B. Fingar D. C. (2012). Regulation and function of ribosomal protein S6 kinase (S6K) within mTOR signalling networks. *Biochem. J.* 441 1–21. 10.1042/bj20110892 22168436

[B46] Meroni P. L. Chighizola C. B. Rovelli F. Gerosa M. (2014). Antiphospholipid syndrome in 2014: more clinical manifestations, novel pathogenic players and emerging biomarkers. *Arthritis Res. Ther.* 16:209. 10.1186/ar4549 25166960 PMC4060447

[B47] Miranda S. Billoir P. Damian L. Thiebaut P. A. Schapman D. Le Besnerais M. (2019). Hydroxychloroquine reverses the prothrombotic state in a mouse model of antiphospholipid syndrome: role of reduced inflammation and endothelial dysfunction. *PLoS One* 14:e0212614. 10.1371/journal.pone.0212614 30870459 PMC6417644

[B48] Miyakis S. Lockshin M. D. Atsumi T. Branch D. W. Brey R. L. Cervera R. (2006). International consensus statement on an update of the classification criteria for definite antiphospholipid syndrome (APS). *J. Thromb. Haemost.* 4 295–306. 10.1111/j.1538-7836.2006.01753.x 16420554

[B49] Morganti C. Bonora M. Ito K. Ito K. (2019). Electron transport chain complex II sustains high mitochondrial membrane potential in hematopoietic stem and progenitor cells. *Stem Cell Res.* 40:101573. 10.1016/j.scr.2019.101573 31539857 PMC6802285

[B50] Mu F. Jiang Y. Ao F. Wu H. You Q. Chen Z. (2020). RapaLink-1 plays an antithrombotic role in antiphospholipid syndrome by improving autophagy both in vivo and vitro. *Biochem. Biophys. Res. Commun.* 525 384–391. 10.1016/j.bbrc.2020.02.084 32093890

[B51] Mulla M. J. Weel I. C. Potter J. A. Gysler S. M. Salmon J. E. Peraçoli M. T. (2018). Antiphospholipid Antibodies Inhibit Trophoblast Toll-Like Receptor and Inflammasome Negative Regulators. *Arthrit. Rheumatol.* 70 891–902. 10.1002/art.40416 29342502 PMC5984662

[B52] Oaks Z. Winans T. Caza T. Fernandez D. Liu Y. Landas S. K. (2016). Mitochondrial dysfunction in the liver and antiphospholipid antibody production precede disease onset and respond to rapamycin in lupus-prone mice. *Arthritis Rheumatol.* 68 2728–2739. 10.1002/art.39791 27332042 PMC5083168

[B53] Perez-Sanchez C. Ruiz-Limon P. Aguirre M. A. Bertolaccini M. L. Kamashta M. A. Rodriguez-Ariza A. (2012). Mitochondrial dysfunction in antiphospholipid syndrome: implications in the pathogenesis of the disease and effects of coenzyme Q(10) treatment. *Blood* 119 5859–5870. 10.1182/blood-2011-12-400986 22529290

[B54] Petri M. (2000). Epidemiology of the antiphospholipid antibody syndrome. *J. Autoimmun.* 15 145–151. 10.1006/jaut.2000.0409 10968901

[B55] Poredos P. Jezovnik M. K. (2018). *Endothelial Dysfunction and Venous Thrombosis.* Los Angeles: SAGE Publications. 10.1177/0003319717732238 28954526

[B56] Possomato-Vieira J. S. Khalil R. A. (2016). Mechanisms of Endothelial Dysfunction in Hypertensive Pregnancy and Preeclampsia. *Adv. Pharmacol.* 77 361–431. 10.1016/bs.apha.2016.04.008 27451103 PMC4965238

[B57] Qian M. Fang X. Wang X. (2017). Autophagy and inflammation. *Clin. Transl. Med.* 6:24. 28748360 10.1186/s40169-017-0154-5 PMC5529308

[B58] Rajendran P. Rengarajan T. Thangavel J. Nishigaki Y. Sakthisekaran D. Sethi G. (2013). The vascular endothelium and human diseases. *Int. J. Biol. Sci.* 9 1057–1069. 10.7150/ijbs.7502 24250251 PMC3831119

[B59] Ramesh S. Morrel C. N. Tarango C. Thomas G. D. Yuhanna I. S. Girardi G. (2011). Antiphospholipid antibodies promote leukocyte-endothelial cell adhesion and thrombosis in mice by antagonizing eNOS via β2GPI and apoER2. *J. Clin. Invest.* 121 120–131. 10.1172/JCI39828 21123944 PMC3007129

[B60] Raschi E. Chighizola C. B. Grossi C. Ronda N. Gatti R. Meroni P. L. (2014). beta2-glycoprotein I, lipopolysaccharide and endothelial TLR4: three players in the two hit theory for anti-phospholipid-mediated thrombosis. *J. Autoimmun.* 55 42–50. 10.1016/j.jaut.2014.03.001 24685231

[B61] Ratzinger F. Panic T. Haslacher H. Perkmann T. Schmetterer K. G. Belik S. (2017). Testing lupus anticoagulants in a real-life scenario - a retrospective cohort study. *Biochem. Med.* 27:030705. 10.11613/BM.2017.030705 28900368 PMC5575653

[B62] Ripoll V. M. Pregnolato F. Mazza S. Bodio C. Grossi C. McDonnell T. (2018). Gene expression profiling identifies distinct molecular signatures in thrombotic and obstetric antiphospholipid syndrome. *J. Autoimmun.* 93 114–123. 10.1016/j.jaut.2018.07.002 30033000 PMC6123515

[B63] Rodríguez-Almaraz M. Herraiz I. Gómez-Arriaga P. Vallejo P. Gonzalo-Gil E. Usategui A. (2018). The role of angiogenic biomarkers and uterine artery Doppler in pregnant women with systemic lupus erythematosus or antiphospholipid syndrome. *Preg. Hypertens.* 11 99–104. 10.1016/j.preghy.2018.01.008 29523283

[B64] Ryter S. W. Bhatia D. Choi M. E. (2019). Autophagy: a Lysosome-Dependent Process with Implications in Cellular Redox Homeostasis and Human Disease. *Antioxid. Redox. Signal.* 30 138–159. 10.1089/ars.2018.7518 29463101 PMC6251060

[B65] Sarbassov D. D. Ali S. M. Sabatini D. M. (2005). Growing roles for the mTOR pathway. *Curr. Opin. Cell Biol.* 17 596–603. 10.1016/j.ceb.2005.09.009 16226444

[B66] Schieke S. M. Phillips D. McCoy J. P. Jr. Aponte A. M. Shen R. F. Balaban R. S. (2006). The mammalian target of rapamycin (mTOR) pathway regulates mitochondrial oxygen consumption and oxidative capacity. *J. Biol. Chem.* 281 27643–27652. 10.1074/jbc.M603536200 16847060

[B67] Schreiber K. Sciascia S. De Groot P. G. Devreese K. Jacobsen S. Ruiz-Irastorza G. (2018). Antiphospholipid syndrome. *Nat. Rev. Dis. Primers* 4 1–20. 29368699 10.1038/nrdp.2018.5

[B68] Tektonidou M. G. Andreoli L. Limper M. Amoura Z. Cervera R. Costedoat-Chalumeau N. (2019). EULAR recommendations for the management of antiphospholipid syndrome in adults. *Ann. Rheum. Dis.* 78 1296–1304. 10.1136/annrheumdis-2019-215213 31092409 PMC11034817

[B69] Turrent-Carriles A. Herrera-Felix J. P. Amigo M. C. (2018). Renal Involvement in Antiphospholipid Syndrome. *Front. Immunol.* 9:1008. 10.3389/fimmu.2018.01008 29867982 PMC5966534

[B70] Velásquez M. Granada M. A. Galvis J. C. Alvarez A. M. Cadavid Á (2019). Estrés oxidativo en células endoteliales inducido por el suero de mujeres con diferentes manifestaciones clínicas del síndrome antifosfolípido. *Biomédica* 39 673–688. 10.7705/biomedica.4701 31860179 PMC7363350

[B71] Widlansky M. E. Malik M. A. (2015). “Vascular Endothelial Function” in *PanVascular Medicine.* ed. Lanzer P. (Berlin: Springer). 89–129.

[B72] Wullschleger S. Loewith R. Hall M. N. (2006). TOR signaling in growth and metabolism. *Cell* 124 471–484. 10.1016/j.cell.2006.01.016 16469695

[B73] Xia L. Zhou H. Wang T. Xie Y. Wang X. Yan J. (2017). Activation of mTOR is involved in anti-β2GPI/β2GPI-induced expression of tissue factor and IL-8 in monocytes. *Thromb. Res.* 157 103–110. 10.1016/j.thromres.2017.05.023 28734155

[B74] Yim W. W. Mizushima N. (2020). Lysosome biology in autophagy. *Cell Discov.* 6:6. 10.1038/s41421-020-0141-7 32047650 PMC7010707

[B75] Yu L. Chen Y. Tooze S. A. (2018). Autophagy pathway: cellular and molecular mechanisms. *Autophagy* 14 207–215. 10.1080/15548627.2017.1378838 28933638 PMC5902171

[B76] Ziporen L. Goldberg I. Arad M. Hojnik M. Ordi-Ros J. Afek A. (1996). Libman-Sacks endocarditis in the antiphospholipid syndrome: immunopathologic findings in deformed heart valves. *Lupus* 5 196–205. 10.1177/096120339600500306 8803890

[B77] Zorova L. D. Popkov V. A. Plotnikov E. Y. Silachev D. N. Pevzner I. B. Jankauskas S. S. (2018). Mitochondrial membrane potential. *Anal. Biochem.* 552 50–59. 10.1016/j.ab.2017.07.009 28711444 PMC5792320

[B78] Zussman R. Xu L. Y. Damani T. Groom K. M. Chen Q. Seers B. (2020). Antiphospholipid antibodies can specifically target placental mitochondria and induce ROS production. *J. Autoimmun.* 111:102437. 10.1016/j.jaut.2020.102437 32224053

