# Supplementary material for: Antiphospholipid Antibodies From Women With Pregnancy Morbidity and Vascular Thrombosis Induce Endothelial Mitochondrial Dysfunction, mTOR Activation, and Autophagy
Source: Front Physiol. 2021 Nov 29;12:706743. doi: 10.3389/fphys.2021.706743 (PMC8667788; doi:10.3389/fphys.2021.706743)
Supplement: Supplementary file 1 [file Data_Sheet_1.PDF]

Supplementary Material

Supplementary Figures

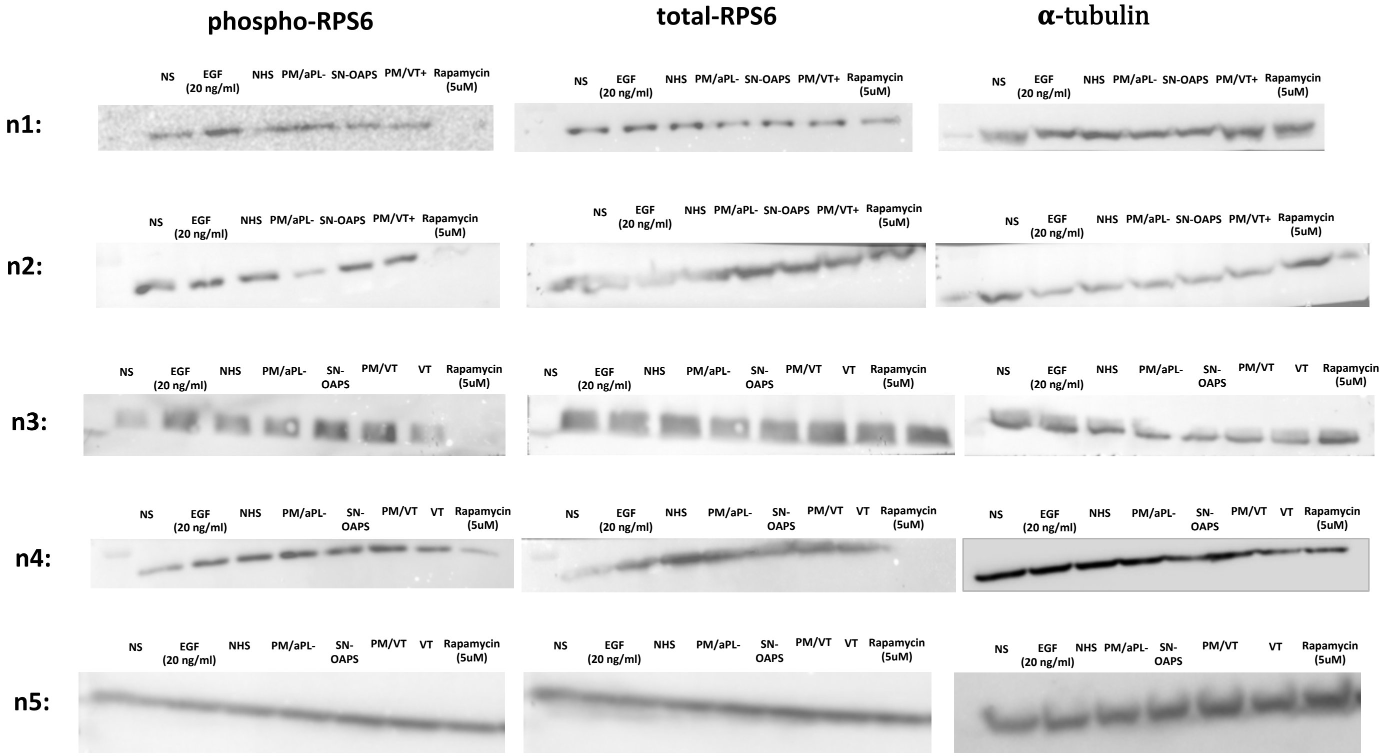

Supplementary Figure 1. Western blotting assays for protein expression of the total and phosphorylated forms of the effector ribosomal protein S6 (RPS6). HUVEC were stimulated with 250 µg/mL polyclonal IgG from women with clinical features of APS and control groups. Epidermal growth factor (EGF) and Rapamycin were used as negative and positive controls respectively, for mTOR inhibition. Non-stimulated cells (NS) were also evaluated as basal control.

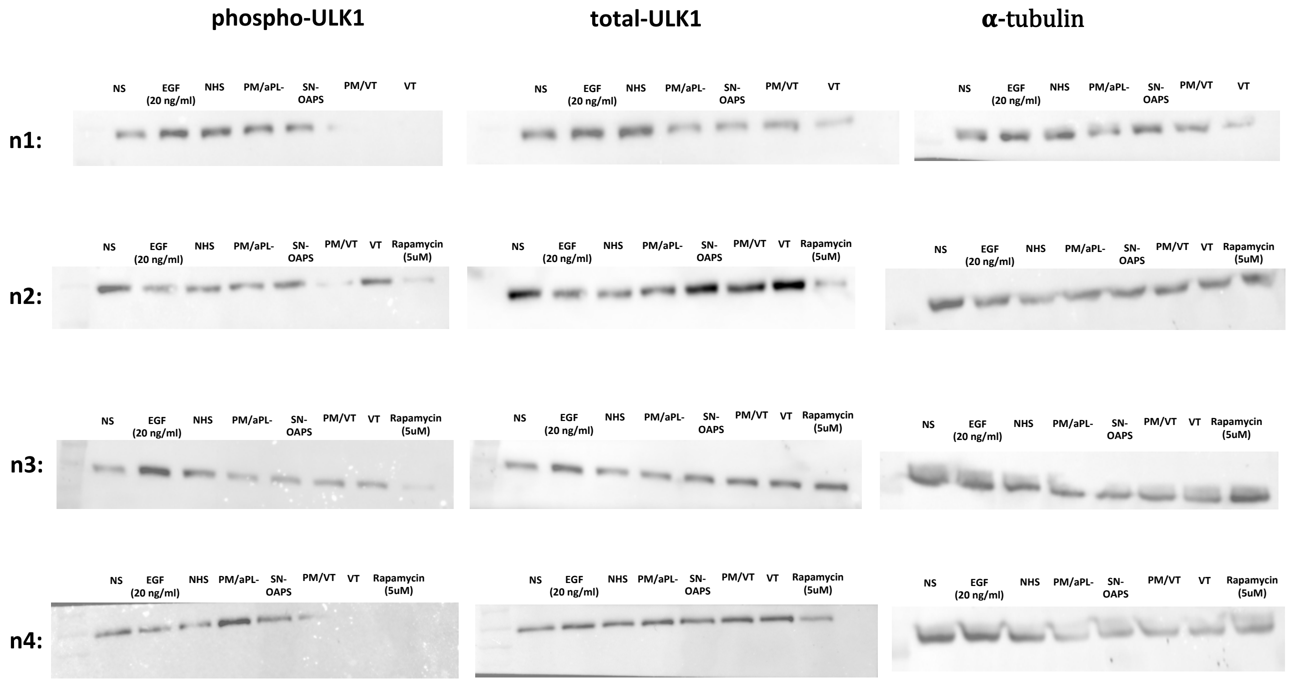

Supplementary Figure 2. Western blotting assays for protein expression of the total and phosphorylated forms of the early autophagy marker ULK1. HUVECs were stimulated with 250 µg/mL polyclonal IgG from women with clinical features of APS and control groups. Epidermal growth factor (EGF) and Rapamycin were used as negative and positive controls, respectively, for autophagy induction. Non-stimulated cells (NS) were also evaluated as basal control.

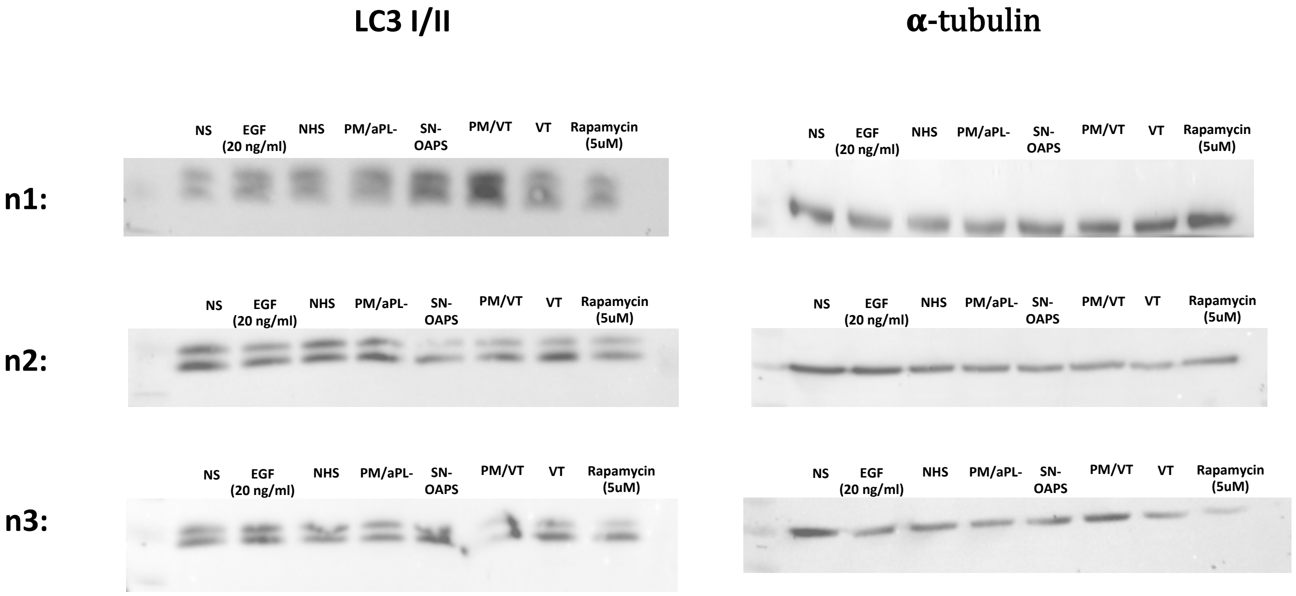

Supplementary Figure 3. Western blotting assays for protein expression of the late autophagy marker LC3-I and LC3-II. HUVEC were stimulated with 250 µg/mL polyclonal IgG from women with clinical features of APS and control groups. Epidermal growth factor (EGF) and Rapamycin were used as negative and positive controls respectively, for autophagy induction. Non-stimulated cells (NS) were also evaluated as basal control.

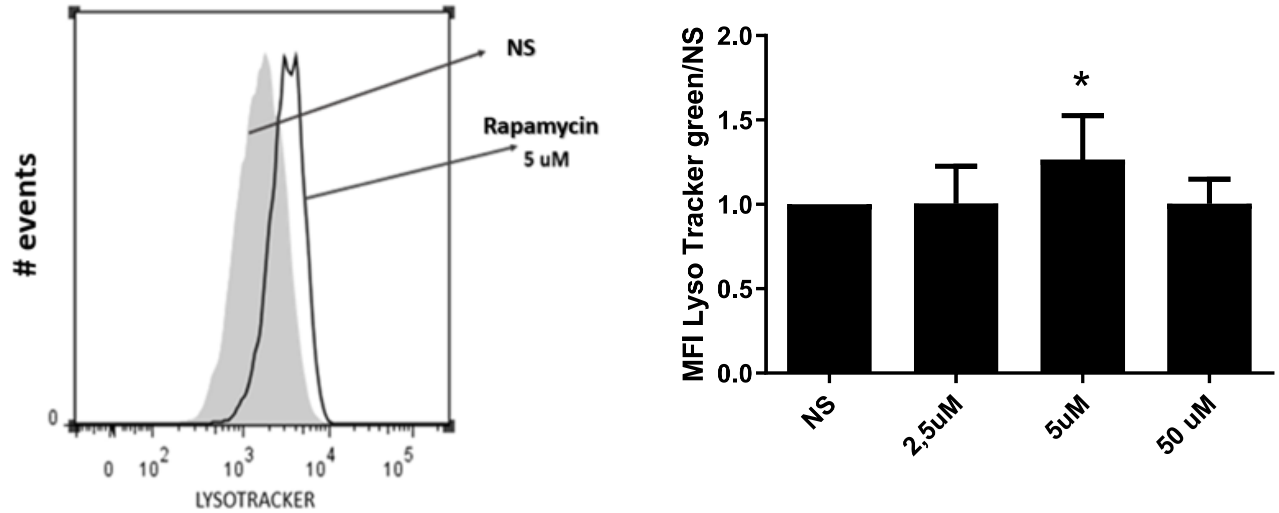

Supplementary Figure 4. Lysosomal acidification assessed with LisoTracker Green and flow cytometric analysis. Representative histogram showing HUVECs treated with 5 μM Rapamycin and non-stimulated cells (NS). Chart shows the levels of LisoTracker Green as MFI and that treatment of HUVECs with 5 μM Rapamycin significantly increased lysosomal acidification. n=5; *p<0.05 *vs* NS as determined by One-way ANOVA and Dunn's post test.
